# Risk-based prioritization of pharmaceuticals in the natural environment in Iraq

**DOI:** 10.1007/s11356-016-6679-0

**Published:** 2016-04-30

**Authors:** Omar S. A. Al-Khazrajy, Alistair B. A. Boxall

**Affiliations:** Environment Department, University of York, Heslington, Wentworth Way, York, YO10 5NG UK

**Keywords:** Pharmaceuticals, Prioritization, Ecotoxicity, Antibiotic resistance, Risk characterization ratio

## Abstract

Numerous studies have demonstrated the occurrence of pharmaceuticals in the natural environment, raising concerns about their impact on non-target organisms or human health. One region where little is known about the exposure and effects of pharmaceuticals in the environment is Iraq. Due to the high number of pharmaceuticals used by the public health sector in Iraq (hospitals and care centres) and distributed over the counter, there is a need for a systematic approach for identifying substances that should be monitored in the environment in Iraq and assessed in terms of environmental risk. In this study, a risk-based prioritization approach was applied to 99 of the most dispensed pharmaceuticals in three Iraqi cities, Baghdad, Mosul and Basrah. Initially, information on the amounts of pharmaceuticals used in Iraq was obtained. The top used medicines were found to be paracetamol, amoxicillin and metformin with total annual consumption exceeding 1000 tonnes per year. Predicted environmental concentrations (PECs) and predicted no-effect concentrations (PNECs), derived from ecotoxicological end-points and effects related to the therapeutic mode of action, were then used to rank the pharmaceuticals in terms of risks to different environmental compartments. Active pharmaceutical ingredients used as antibiotics, antidepressants and analgesics were identified as the highest priority in surface water, sediment and the terrestrial environment. Antibiotics were also prioritized according to their susceptibility to kill or inhibit the growth of bacteria or to accelerate the evolution and dissemination of antibiotic-resistant genes in water. Future work will focus on understanding the occurrence, fate and effects of some of highly prioritized substances in the environment.

**Electronic supplementary material:**

The online version of this article (doi:10.1007/s11356-016-6679-0) contains supplementary material, which is available to authorized users.

## Introduction

It is estimated that more than 1500 active pharmaceutical ingredients (APIs) are currently in use. Following use, these compounds can be emitted into the natural environment e.g. via wastewater collection and treatment networks (Boxall et al. 2012; Ginebreda et al. 2010). The ongoing use of many of these APIs by society means that the active substances and their major metabolites will occur in the environment continuously (Monteiro and Boxall 2010).

For most pharmaceuticals in use, the evidence that they have deleterious effects on the natural environment is still limited and our knowledge of the fate of these pharmaceuticals in the environment is still deficient (Roos et al. 2012). This is partly due to the fact that the number of APIs in use is large and that experimental data on the environmental levels, fate and effects are available for only a small proportion of these substances. For example, the knowledge of environmental exposure to antibiotics which may lead to possible evolution and dissemination of antibiotic-resistant pathogens in bacteria is limited (Bengtsson-Palme and Larsson 2016). To experimentally assess the environmental risk of all APIs in use would be a challenge (Perazzolo et al. 2010). One solution is to use formalized prioritization procedures that identify those substances in use that pose the greatest risks towards the natural environment (Boxall et al. 2012). By using these approaches, experimental testing resources can then be focused on those substances that are likely to have the greatest impact.

Several studies have been recently performed that employ different approaches for ranking and assessing the risk posed by APIs to the environment. Most have focused on surface or drinking water and the risks to aquatic organisms or human health. These approaches have been applied in Switzerland (Perazzolo et al. 2010), USA (Kostich and Lazorchak 2008; Dong et al. 2013), France (Besse and Garric 2008), the UK (Boxall et al. 2003; Guo et al. 2016), South Korea (Kim et al. 2008) and Sweden (Roos et al. 2012). Many of these approaches use exposure and toxicological predictions so they can be readily applied to large numbers of compounds with limited data (Boxall et al. 2012).

Most prioritization studies have focused on North America and Western Europe, so our knowledge of priorities in other geographical areas such as Eastern Europe, Africa, Asia and South America is limited. This can be partly explained by the challenges in obtaining information on API usage in these regions. Moreover, although there are strong incentives to introduce the evaluation of an antibiotic to select for resistance into environmental risk assessment guidelines (Bengtsson-Palme and Larsson 2016), none of the previous prioritization approaches has attempted to assess the risk of antibiotics in the environment in terms of their potential to select for antimicrobial resistance.

In Iraq, there are no specific management guidelines for pharmaceuticals in the environment. Pharmaceuticals are freely available to everyone without any restriction and regulation or even without prescription, and there are many routes by which these substances are distributed to the population. One route is the public health sector which is represented by the Ministry of Health (MOH) via the state company for importation and distribution of drug and medical appliances (KIMADIA). The second source is the private sector (licensed and unlicensed low value manufacturers) which includes 23 manufacturing plants, importers and dispensers who supply the local markets with unknown quantities of pharmaceuticals. Additionally, all the locally produced and imported finished pharmaceuticals are not subjected to taxes in order to make them affordable for most of the population (USAID; 2007; EMRO WHO 2011; MOH 2011).

With a highly urbanized population, Iraq still has insufficient environmental management and suffers from poor and old water distribution systems and contaminated main water resources (UNEP 2003). Due to the absence of water quality regulations and the continuous discharges from industry and households via insufficient wastewater treatment plants (WWTPs), up to 19 % of the Iraqi population is exposed to unsafe water (UNEP 2003; USAID 2007). In addition, only 32 % of the population is served with wastewater treatment, meaning that a significant amount of untreated wastewater is released to the environment (COSIT 2012). Few studies to evaluate the quality of environmental systems in Iraq have been performed, and most that have been performed have focused on monitoring the occurrence of trace metals, polycyclic aromatic hydrocarbons (PAHs) and non-polar lipids in the aquatic environment (Abaychi and DouAbul 1985; Al-Saad 1987; Rushdi et al. 2014). The risks of emerging contaminants such as pharmaceuticals have been neglected.

The aim of the present study was therefore to establish the importance of API exposure as a pressure on the natural environment in Iraq and to identify APIs of most concern in local aquatic and terrestrial environments of the three main cities in the country (Baghdad, Mosul and Basrah), where only little is currently known about the exposure and effects of these substances. The prioritization approaches used to achieve this were based on the potential for APIs to enter the aquatic and terrestrial environments and their potential toxic effects on the ecosystems, bacterial community and human health.

## Materials and methods

### Prioritization approach

**Fig. 1:**
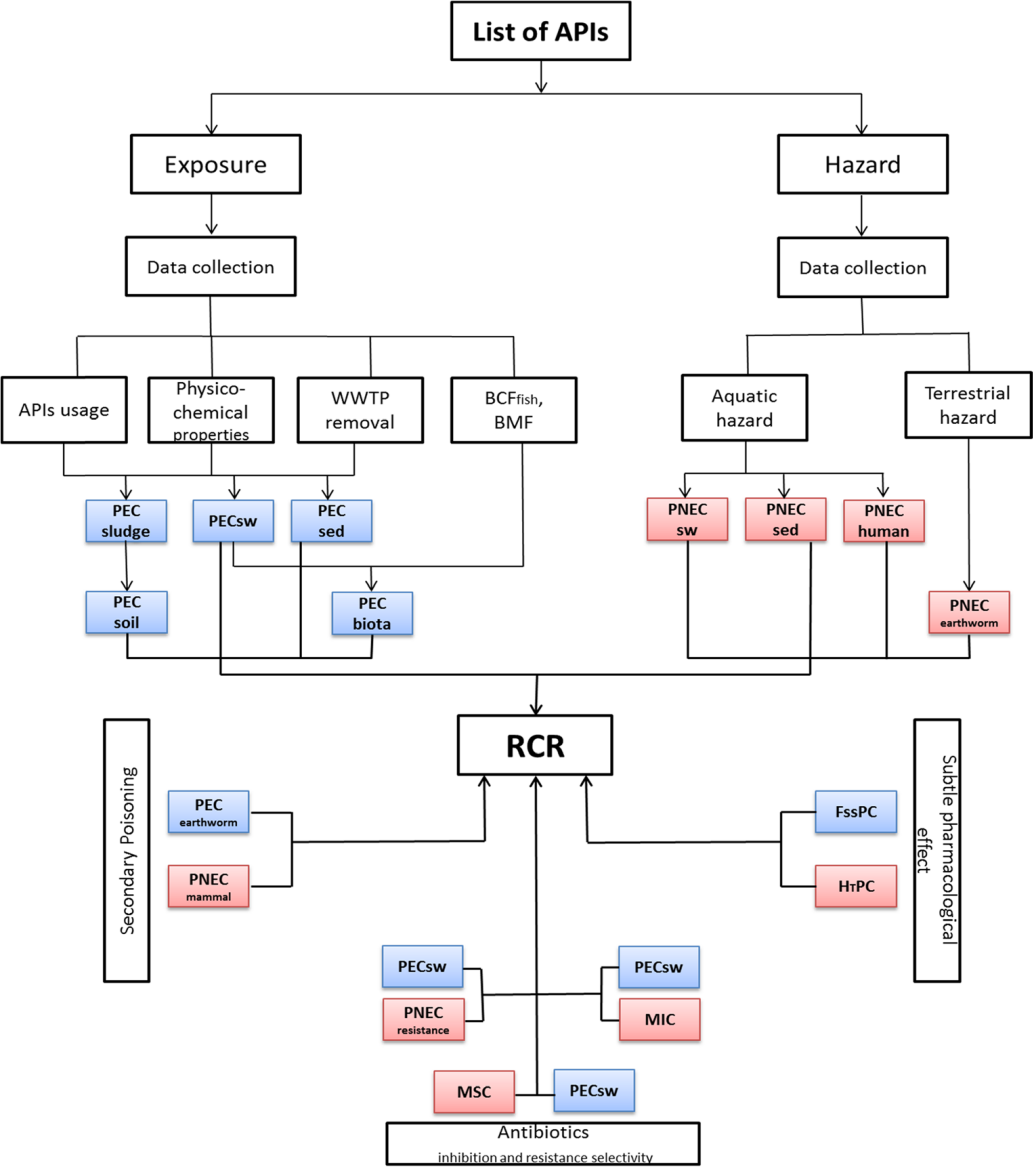
The developments of prioritization approach of pharmaceuticals in the environment in Iraq. *RCR* risk characterization ratios, *PECsw*, *PNECsw* predicted environmental concentration and predicted no-effect concentration in surface water, *PECsludge* predicted environmental concentration in sludge compartment, *PECsed*, *PNECsed* predicted environmental concentration and predicted no-effect concentration in sediment compartment, *WWTP*wastewater treatment plant, *BCF* fish bioconcentration factor, *BMF* biomagnification factor, *PECbiota* predicted environmental concentration in biota (e.g. fish), *PNEChuman* predicted no-effect concentration in humans from drinking water and fishery products consumption, *PECsoil* predicted environmental concentration in soil, *FSSPC* fish steady state plasma concentration, *H*_*T*_ *PC* human therapeutic plasma concentration, *PECwarthworm* predicted environmental concentration in earthworm, *PNECearthworm* predicted no-effect concentration in earthworm, *PNECmammal* predicted no effect concentration in mammal, *MIC* minimal inhibitory concentration, *PNECresistance* predicted no-effect concentration for antibiotics resistance selection, *MSC* minimal selective concentration

The prioritization approach is illustrated in Fig. 1 and involved the use of predicted environmental concentrations (PECs) and concentrations relating to different effect endpoints (i.e. predicted no-effect concentrations (PNECs), human plasma therapeutic concentrations (H_T_PCs), minimal inhibitory concentrations (MICs) and minimal selective concentrations (MSCs)) for each of the pharmaceuticals in aquatic and terrestrial systems. PECs and PNECs were then used to calculate risk characterization ratios (RCRs) for apical endpoints, secondary poisoning, toxicity to humans and antimicrobial resistance selection. Pharmaceuticals were then ranked based on their RCRs where compounds with the highest RCRs were considered the highest priority.

### Data collection

#### Usage data

Data on the consumption of pharmaceuticals for hospitals and primary care centres in Iraq in 2014 were obtained from the state company KIMADIA (Kimadia, access 2014). To obtain the total amount of pharmaceuticals consumed, concentrations of active ingredient in packaging units (i.e. blister, bottle, etc.) were converted into mass units. Vitamins, medical supplements, electrolytes and vaccines were excluded which reduced the list of APIs to 99 compounds. In the case of combined medicines, only individual active ingredients were considered and summed up to calculate the weight of pharmaceutical compound.

Information is scarce on the use of over-the-counter pharmaceuticals in Iraq. However, research by the Center of Market Research and Consumer Protection at the University of Baghdad (Mohammed et al. 2009) indicates that over-the-counter usage can contribute 68 % of the total usage of pharmaceuticals in Iraq. Therefore, to obtain a total pharmaceutical usage in Iraq (for both hospitals and primary care centres and over the counter), the results of the analysis of the KIMADIA data were multiplied by a factor of 3.125. Some APIs, such as cancer treatments or those used in surgical procedures in hospitals, were not corrected (multiplied by the factor) as they would not be distributed over the counter. The final usage data are provided in the supporting information (Table S1).

#### Effects data and physico-chemical properties

To estimate the environmental risk posed by the pharmaceuticals to aquatic and terrestrial ecosystems in Iraq, data on toxicity of the APIs to algae, daphnia, fish and earthworms was used. The data collection included acute and chronic ecotoxicity endpoints (typically the most sensitive LC/EC50 value). These data were obtained from the peer-reviewed literature, grey literature and available online databases (e.g. Swedish voluntary environmental classification of pharmaceuticals at www.fass.se). As experimental ecotoxicity data were not available for a large number of the pharmaceuticals, estimation tools, such as Quantitative Structure-Property Relationships (QSAR) used in the Organisation for Economic Co-operation and Development (OECD QSAR, 2013) Toolbox and the Ecological Structure Activity Relationship ECOSAR (USEPI 4.1) software, were used to fill data gaps (Guo et al. 2016). The database present in the QSAR Toolbox was used to identify experimental data for molecules deemed ‘similar’ to each of the individual pharmaceutical with no data. Then, within the software, a relationship was built to allow an estimation of the ecotoxicological endpoint for the query molecule. Regarding human and mammalian toxicity effects from oral exposure, endpoints such as acceptable daily intake (ADI) values and median lethal dose (LD50) for rat/mouse were used (Technical Guidance for Deriving Environmental Quality Standards EC 2011, Carvalho et al. 2015; Guo et al. 2016). The H_T_PCs available in peer-reviewed publications were used in the fish plasma model. Finally, for terrestrial toxicity, earthworm acute toxicity (14-day LC50 in mM kg^−1^ dry soil) was predicted using the QSAR available in ECOSAR for compounds with no experimentally determined earthworm ecotoxicity data. Due to the absence of experimentally determined effects of antibiotics in complex microbial communities, the theoretical MICs, MSCs and PNECs selective resistance calculated by Bengtsson-Palme and Larsson (2016) were used.

Physico-chemical properties required for predicting the fate and behavior of pharmaceuticals in the environment were collated from published articles and open resources. DrugBank, NCCOS (2014) was used to obtain acid dissociation constants (pKa), and the CODATA (2014) database was used to obtain octanol-water partition coefficients (Kow). As there was a lack of experimental data on organic carbon partition coefficient (Koc) for the APIs, for compounds where experimental Koc data were not available, we used the estimation model developed by Franco and Trapp (2008). Excretion profiles for pharmaceuticals were obtained from the peer-reviewed literature, databases or pharmaceutical safety data sheets (i.e. MEDSAFE, Pfizer).

#### Wastewater generation and dilution factor

Information on wastewater disposal for the main highly urbanized cities in Iraq (Baghdad, Mosul and Basrah) was collected. The daily generated wastewater discharges are 1.6 million m^3^/day in Baghdad, 0.5 million m^3^/day in Mosul and 0.331 million m^3^/day in Basrah (COSIT 2014). These data were used to calculate the wastewater generated per inhabitant (Supporting Information, Equation S1).

It is difficult to determine the dilution factor (DF) in countries with none or very scarce hydrological information like Iraq. For this purpose, we therefore used two dilution factors of 10 and 40 which had been estimated based on a national scale for Iraq by Keller et al. (2014). The percentage of wastewater treatment efficiency will also be important for the calculation of exposure concentrations in surface water so information was also collected on the percentage connectivity to wastewater treatment plants for the three cities. Data on the population, wastewater per capita, wastewater treatment percentage and dilution factors for the cities under study is provided in Table S3 in the supporting information.

### Exposure assessment

Predicted environmental concentrations of APIs on the usage list were calculated in aquatic systems (surface water and sediment) and terrestrial systems according to the Guideline on the Environmental Risk Assessment of Medicinal Products for Human Use (EMEA 2006) and the Technical Guidance Document on Risk Assessment part II (TGD 2003) with some modifications to be fitted to the case of Iraq. In surface water, PECsw values for APIs were calculated using the following equation (Eq.1):

1 $$ \mathrm{PECsw}=\frac{\mathrm{Subinhab}\times \mathrm{Fexc}}{\mathrm{WasteWinhab}\times \mathrm{Dilution}}\times \left(1-\frac{\mathrm{Sludge}\ \mathrm{inhab}\times \mathrm{K}\mathrm{o}\mathrm{c}\times \mathrm{focsludge}}{\mathrm{WasteWinhab}+\left(\mathrm{Sludge}\ \mathrm{inhab}\times \mathrm{K}\mathrm{o}\mathrm{c}\times \mathrm{focsludge}\right)}\right) $$

where PEC_SW_ is the PEC of an API in surface water. Sub_inhab_ is the consumed amount of pharmaceuticals per inhabitant in Iraq per day (mg/inh/day) and was calculated based on annual pharmaceutical consumption (kg year^−1^) and using the population of Iraq (34.2 million), (Eq. S2, Supporting Information); DF is the dilution factor of 10 and 40 and WW_inhab_ is the daily amount of wastewater per inhabitant in either Baghdad, Mosul or Basrah. F_exc_ is the fraction of parent ingredients excreted unchanged via human metabolism. Sludge_inhab_ [kg/inh/day] is the mass of waste sludge per inhabitant per day, which is 0.074 (EC 2001); Koc is the organic carbon partitioning coefficient determined experimentally or estimated according to Franco and Trapp (2008) for ionisable chemicals using Kow and pKa; and foc_sludge_ is the fraction of sludge organic carbon and was assumed to be 0.326 (Struijs et al. 1991).

The assumption of removal by adsorption was just used in the case of Baghdad because of the absence of wastewater treatment in both Mosul and Basrah. For Mosul, there are no wastewater treatment plants in the city while for Basrah the efficiency percentage of wastewater treatment in existing WWTPs is zero (COSIT 2014).

For sediment, the standard algorithms in the TGDII (2003) was used to estimate concentrations of the APIs in terms of wet weight (ww) (PECsed_ww), and since the final PECsed was calculated in terms of dry weight, a conversion step was applied to determine PECsed on a dry weight basis (Carvalho et al. 2015) (Eqs. S2 to S5 in Supporting Information).

For the calculation of the PECbiota, the following equation was used.

2 $$ \mathrm{PECbiota} = \mathrm{PECsw} \times \mathrm{B}\mathrm{CFbiota} \times \mathrm{B}\mathrm{M}\mathrm{F} $$

where BCF is the bioconcentration factor for biota (e.g. fish) which was retrieved when available or calculated according to Fick et al. (2010) (Eq. S7, Supporting Information). Default biomagnification factor (BMF) values were retrieved from technical guidance document (EC 2001).

Data on measured levels of pharmaceuticals in fish plasma following exposure via water are still scarce (Fick et al. 2010). As an indicator of specific drugs’ potential to cause adverse pharmacological effects at certain concentrations, the fish steady state plasma concentration (FSSPC) resulting from exposure via surface water was calculated (Eq. S8 in Supporting Information). Predictions were based on estimations of the partitioning of an API between the aqueous phase and arterial blood in the fish (Pblood/water) (Eqs. S9 and S10 in Supporting Information). This partition coefficient was initially estimated based on the log K_OW_ of the API, and this was subsequently combined with the PECsw to estimate the FSSPC.

PECsoil was calculated since a PECsludge had been calculated using algorithms described in the TGD (2003). To estimate the concentration of an API in earthworms (PECearthworm), the concentration in the earthworms on a wet weight basis (C earthworm) was calculated using an estimate of the concentration in porewater (Cporewater) from PECsoil by considering the partitioning behaviour of substances between the soil and aqueous phase (Eqs. S11 to S13 in Supporting Information). The BCF for earthworms was calculated according to the approach in the TGD (2003).

### Hazard characterization

In order to calculate PNECs for toxicity to surface water organisms, effects data were divided by a relevant assessment factor (AF), i.e. acute QSAR data =1000; acute experimental data =100; chronic QSAR data =100 and chronic experimental data =10, (TGD 2003). In instances where more than one ecotoxicological value was found, the most sensitive endpoint was used for the generation of the PNEC. PNECs for earthworms were obtained by dividing the 14-day LC50 value by an AF of 1000. PNECs for mammals were obtained by dividing median lethal doses for mouse or rat by an AF of 100. PNECs for resistance were obtained from MSCs using an AF of 10. AFs were not used for the estimation of concentrations causing mode of action-based effects (using the H_T_PC) or for the MICs for microbes. Specific equations are provided in the Supporting Information (Eqs. S9–S11).

## Results and discussion

### Experimental data availability

Experimental acute ecotoxicological data were only available for 51 of the 99 APIs under consideration. Chronic ecotoxicity endpoints were only available for 21 compounds so the ecotoxicity values of the others were estimated using the QSAR Toolbox and the ECOSAR software. In terms of data on mammalian safety, data were available on the toxicity of 72 compounds, 87 had an ADI and 88 had a H_T_PC. Experimental bioconcentration factors in fish (BCFfish) were only available for two compounds (diclofenac and naproxen). Experimental organic carbon partition coefficient (Koc) values were only available for 21 pharmaceuticals (Table 1).

**Table 1:** Summary of experimental data available for the APIs under consideration

Parameter	Number of compounds
Excretion profile (Fex)	81
Log Kow	90
pKa	94
Experimental Koc	21
Experimental endpoint (acute LC(EC) 50)	51
Experimental endpoint (chronic LC(EC) 50)	21
Experimental Bioconcentration factor in fish	2
Acceptable daily intake (ADI)	87
Mammalian toxicity (LD50) for rat/mouse	72
Human therapeutic plasma concentration (H_T_PC)	88

*Fex* fraction of parent ingredients excreted unchanged via human metabolism, *log Kow* octanol-water partitioning coefficient, *pKa* dissociation coefficient, *Koc* organic carbon partition coefficient, *EC50* 50 % effective concentration, *LC50* 50 % lethal concentration, *LD50* median lethal dose for rat/mouse, *BCF* bioconcentration factor in fish, *ADI* acceptable daily intake, *H*_*T*_ *PC* human therapeutic plasma concentration

### RCR lists of APIs in different systems

The top ranked APIs with RCR >0.1, derived from the different prioritizations for the aquatic environments in the three cities under consideration and at two dilution factors, are presented in Tables 2 and 3 for surface water and Table 4 for sediment. The compounds on the top of the prioritization list with an RCR ≥1 according to PECsw and acute ecotoxicological endpoint were amoxicillin, azithromycin, cefalexine, valproic acid, erythromycin, paracetamol and clarithromycin in Mosul and Basrah. In Baghdad, only five compounds had an RCR ≥1 (amoxicillin, clarithromycin, azithromycin, valproic acid and paracetamol). This difference between the cities is due to the absence of wastewater treatment processes in Mosul and Basrah and hence that no removal of APIs by adsorption on sludge will occur in these cities. When chronic effects were considered, at the lower dilution factor, six compounds had RCR values ≥1 for all cities i.e. amoxicillin, clarithromycin, diclofenac, miconazole nitrate and mefenamic acid. At the higher dilution rate, only two compounds (amoxicillin and clarithromycin) had an RCR ≥1 (Table 3). All other pharmaceuticals had a risk score <0.1 (Table S3, Supporting Information).

**Table 2:** Top ranked APIs with RCR >0.1 from each prioritization approach for exposure via surface water at D = 10

Location	RCR	Low level trophic		Subtle effects on fish	Mammalian predator	Human (uptake from fishery products)	Human (uptake from drinking water)	Effect of antibiotics on bacteria		
		Acute aquatic	Chronic aquatic	FSSPC: H_T_PC	PECFISH: PNEC mammal	PECFISH: PNEC biota, hh	PECSW: PNEC dw, hh	PECSW: MIC	PECSW: MSC	PEC SW: PNEC resistance selection
		(PECSW: acute PNEC aquatic)	(PECSW: chronic PNEC aquatic)							
		D10	D10	D10	D10	D10	D10/D40	D10	D10	D10
Baghdad	>10	Amoxicillin Clarithromycin	Amoxicillin Clarithromycin	Phenylephrine Atorvastatin Mebeverine Mefenamic acid	Phenylephrine	Phenylephrine				Amoxicillin Metronidazole
	1–≤10	Azithromycin Valproic acid Paracetamol	Diclofenac Miconazole nitrate Mefenamic acid		Mefenamic acid Miconazole nitrate	Atorvastatin		Amoxicillin	Amoxicillin Ceftriaxone Sodium Metronidazole	Trimethoprim Ceftriaxone Sodium Ampicillin Clarithromycin Cefalexine
	0.1–<1	Cefalexine Ciprofloxacin Miconazole nitrate Mefenamic acid Erythromycin Ibuprofen	Erythromycin Paracetamol Naproxen Azithromycin Mesalazine Mebeverine	Amitriptyline Metformin Miconazole nitrate	Valproic acid Diazepam Atorvastatin	Mefenamic acid Valproic acid Miconazole nitrare		Ceftriaxone Sodium Metronidazole Ampicilline	Clarithromycin Trimethoprim Cefalexine Ampicilline	Ciprofloxacin Azithromycin
Mosul	>10	Amoxicillin Azithromycin	Amoxicillin Clarithromycin	Phenylephrine Atorvastatin Mebeverine	Phenylephrine	Phenylephrine				Amoxicillin Metronidazole
	1–≤10	Ciprofloxacin Valproic acid Erythromycin Paracetamol ClarithromycinCefalexine	Erythromycin Diclofenac Miconazole nitrate Mefenamic acid	Amitriptyline Mefenamic acid		Atorvastatin		Amoxicillin	Amoxicillin Ceftriaxone Sodium Metronidazole	Ciprofloxacin Trimethoprim Ceftriaxone Sodium Ampicillin Clarithromycin Cefalexine Erythromycin
	0.1–<1	Miconazole nitrate Mefenamic acid Ibuprofen Tetracycline Metronidazole Trimethoprim	Paracetamol Azithromycin Naproxen Mesalazine Mebeverine	Metformin Miconazole nitrate	Diazepam Atorvastatin Octreotide	Octreotide	Tramadol	Ceftriaxone Sodium Metronidazole Ciprofloxacin Ampicillin Trimethoprim	Ciprofloxacin Clarithromycin Trimethoprim Cefalexine E ythromycin Ampicillin	Azithromycin
Basrah	>10	Amoxicillin Azithromycin	Amoxicillin Clarithromycin Erythromycin	Phenylephrine Atorvastatin Mebeverine	Phenylephrine	Phenylephrine				Amoxicillin Metronidazole
	1–≤10	Ciprofloxacin Valproic acid Erythromycin Paracetamol Clarithromycin Cefalexine	Diclofenac Miconazole nitrate Mefenamic acid	Amitriptyline Mefenamic acid		Atorvastatin		Amoxicillin	Amoxicillin Ceftriaxone Sodium Metronidazole	Ciprofloxacin Trimethoprim Ceftriaxone Sodium Ampicillin Clarithromycin Cefalexine Erythromycin
	0.1–<1	Miconazole nitrate Mefenamic acid Ibuprofen Tetracycline Metronidazole Trimethoprim Atorvastatin	Paracetamol Azithromycin Naproxen Mesalazine Mebeverine	Metformin Miconazole nitrate Glibenclamide	Diazepam Atorvastatin Octreotide Miconazole nitrare Captopril	Octreotide Captopril	Tramadol	Ceftriaxone Sodium Metronidazole Ciprofloxacin Ampicillin Trimethoprim Erythromycin	Ciprofloxacin Clarithromycin Trimethoprim Cefalexine Erythromycin Ampicillin	Azithromycin

*PECsw* predicted environmental concentration in surface water, *FSSPC* fish steady-state plasma concentration, *H*_*T*_ *PC* human plasma therapeutic concentration, *PECFISH* predicted environmental concentration in fish, *PNEC dw* predicted no-effect concentrations in drinking water, *PNECaquatic/PNECmammal* predicted no-effect concentrations in aquatic and mammalian organisms, *MIC* minimal inhibitory oncentration, *MSC* minimal selective concentration, *PNEC resistance selection* predicted no effect concentrations for antimicrobial resistance, *D* dilution factor

**Table 3:** Top ranked APIs with RCR >0.1 from each prioritization approach for exposure via surface water at D = 40

Location	RCR	Low level trophic		Subtle effects on fish	Mammalian predator	Human (uptake from fishery products)	Human (uptake from drinking water)	Effect of antibiotics on bacteria		
		Acute aquatic	Chronic aquatic	FSSPC: H_T_PC	PECFISH: PNEC mammal	PECFISH: PNEC biota, hh	(PECSW: PNEC dw, hh)	PECSW: MIC	PECSW: MSC	PEC SW: PNEC resistance selection
		(PECSW: acute PNEC aquatic)	(PECSW: chronic PNEC aquatic)							
		D40	D40	D40	D40	D40	D10/D40	D40	D40	D40
Baghdad	>10	Amoxicillin	Amoxicillin	Phenylephrine	Phenylephrine					Amoxicillin Metronidazole
	1–≤10	Clarithromycin Azithromycin Valproic acid	Clarithromycin	Atorvastatin Mebeverine		Phenylephrine			Amoxicillin	Trimethoprim Ceftriaxone Sodium Ampicillin Clarithromycin
	0.1–<1	Paracetamol Cefalexine Ciprofloxacin Miconazole nitrate Mefenamic acid	Diclofenac Miconazole nitrate Mefenamic acid Erythromycin Paracetamol	Mefenamic acid Amitriptyline	Mefenamic acid Miconazole nitrate	Atorvastatin		Amoxicillin	Ceftriaxone Sodium Metronidazole	Cefalexine Ciprofloxacin Azithromycin Ciprofloxacin
Mosul	>10	Amoxicillin	Amoxicillin	Phenylephrine	Phenylephrine	Phenylephrine				Amoxicillin Metronidazole
	1–≤10	Azithromycin Ciprofloxacin Valproic acid Erythromycin	Clarithromycin Erythromycin	Atorvastatin Mebeverine					Amoxicillin	Ciprofloxacin Trimethoprim Ceftriaxone Sodium Ampicillin Clarithromycin Cefalexine Erythromycin
	0.1–<1	Paracetamol Clarithromycin Cefalexine Miconazole nitrate Mefenamic acid	Diclofenac Miconazole nitrate Mefenamic acid Paracetamol Azithromycin Naproxen	Amitriptyline Mefenamic acid		Atorvastatin Octreotide	Tramadol	Amoxicillin Ceftriaxone Sodium	Ceftriaxone Sodium Metronidazole Ciprofloxacin	Azithromycin
Basrah	>10	Amoxicillin	Amoxicillin	Phenylephrine Atorvastatin	Phenylephrine HCL	Phenylephrine				Amoxicillin
	1–≤10	Azithromycin Ciprofloxacin Valproic acid Erythromycin	Clarithromycin Erythromycin	Mebeverine				Amoxicillin	Amoxicillin	M tronidazole Ciprofloxacin Trimethoprim
	0.1–<1	Paracetamol Clarithromycin Miconazole nitrate Cefalexine Mefenamic acid Tetracycline Ibuprofen Diphenhydramine	Diclofenac Miconazole nitrate Mefenamic acid Paracetamol Azithromycin Naproxen	Amitriptyline Mefenamic acid Metformin	Diazepam	Atorvastatin Octreotide	Tramadol	Ceftriaxone Sodium Metronidazole	Ceftriaxone Sodium Metronidazole Ciprofloxacin	Ceftriaxone Sodium Ampicillin Clarithromycin Cefalexine Erythromycin Azithromycin

*PECsw* predicted environmental concentration in surface water, *FSSPC* fish steady-state plasma concentration, *H*_*T*_ *PC* human plasma therapeutic concentration, *PECFISH* predicted environmental concentration in fish, *PNEC dw* predicted no-effect concentrations in drinking water, *PNECaquatic/PNECmammal* predicted no-effect concentrations in aquatic and mammalian organisms, *MIC* minimal inhibitory oncentration, *MSC* minimal selective concentration, *PNEC resistance selection* predicted no effect concentrations for antimicrobial resistance, *D* dilution factor.

**Table 4:** Top ranked APIs with RCR >0.1 in the three cities (Baghdad, Mosul, Basrah) according to the predicted concentrations in sediment (PECsed) and at 10 and 40 dilution factors

	Baghdad				Mosul				Basrah			
	Acute aquatic (PECsed: acute PNECsed)		Chronic aquatic (PECsed: chronic PNECsed)		Acute aquatic (PECsed: acute PNECsed)		Chronic aquatic (PECsed: chronic PNECsed)		Acute aquatic (PECsed: acute PNECsed)		Chronic aquatic (PECsed: chronic PNECsed)	
RCR	D10	D40	D10	D40	D10	D40	D10	D40	D10	D40	D10	D40
>10	Amoxicillin				Amoxicillin Erythromycin Azithromycin Ciprofloxacin	Amoxicillin	Amoxicillin Clarithromycin		Amoxicillin Erythromycin Azithromycin Ciprofloxacin	Amoxicillin Erythromycin	Amoxicillin Clarithromycin	Amoxicillin
1-10	Erythromycin Azithromycin Valproic acid Paracetamol Ciprofloxacin	Amoxicillin Erythromycin Azithromycin Valproic acid	Amoxicillin Clarithromycin Diclofenac Miconazole nitrate Mefenamic acid	Amoxicillin Clarithromycin	Valproic acid Paracetamol Cefalexin	Azithromycin Erythromycin Ciprofloxacin Valproic acid	Erythromycin Diclofenac Miconazole nitrate Mefenamic acid	Amoxicillin Clarithromycin Erythromycin Diclofenac	Valproic acid Paracetamol Clarithromycin	Azithromycin Ciprofloxacin Valproic acid	Erythromycin Diclofenac Miconazole nitrate Mefenamic acid	Clarithromycin Erythromycin
0.1–<1	Cefalexine Miconazole nitrate Clarithromycin Mefenamic acid Ibuprofen Metronidazole Erythromycin	Paracetamo Ciprofloxacin Cefalexine Miconazole nitrate Clarithromycin Mefenamic acid	Paracetamol Naproxen Erythromycin Azithromycin Mesalazine Mebeverine	Diclofenac Miconazole nitrate Mefenamic acidParacetamol	Miconazole nitrate Clarithromycin Mefenamic acid Ibuprofen Tetracycline Metronidazole Trimethoprim	Paracetamol Cefalexine Miconazole nitrate Clarithromycin Mefenamic acid	Paracetamol Azithromycin Naproxen Mesalazine Mebeverine	Miconazole nitrate Mefenamic acid Paracetamol Azithromycin Naproxen	Cefalexine Miconazole nitrate Mefenamic acid Ibuprofen Metronidazole	Paracetamol Clarithromycin Cefalexine Miconazole nitrate Mefenamic acid	Paracetamol Naproxen Azithromycin Mesalazine Mebeverine	Diclofenac Miconazole nitrate Mefenamic acid Paracetamol

The PECsed and PNECsed were calculated with the equilibrium partitioning method from the PECsw and PNECsw, respectively

*PECsed* predicted environmental concentration in sediment, *PNECsed* predicted no effect concentrations in sediment, *D* dilution factor

When the potential impact of subtle pharmacological effects were considered by comparing the human therapeutic concentration in plasma to estimated levels in fish plasma, using a dilution factor of 10, phenylephrine, atorvastatin and mebeverine showed RCR values >1 in all three cities. Additionally, amitriptyline and mefenamic acid had an RCR ≥1 In Mosul and Basrah. Using the higher dilution factor, only phenylephrine showed RCR >1 in Baghdad and Mosul whereas phenylephrine and atorvastatin exceeded an RCR of 1 in Basrah (Table 3).

Assessment of human exposure from consumption of fish products showed that phenylephrine and atorvastatin had an RCR >1 in all cities when a DF of 10 was used and only phenylephrine (RCR >1) when the DF of 40 was used. For human exposure via drinking water, tramadol HCL was the highest ranked compound (with an RCR between 0.1 and 1 while for the rest of pharmaceuticals the RCR was below 0.1.

The predicted concentrations for amoxicillin in all cities when DF = 10 was used were close to the MICs, and the RCRs were between 1 to 10, suggesting that concentration could be high enough to inhibit growth of or kill bacteria. Amoxicillin and metronidazole were on the top list of antibiotics identified as a risk for selection for bacterial resistance (RCR >10), with a further seven APIs having RCR values between 1 and 10 (Tables 2 and 3).

The highest ranked APIs based on acute effect in sediment organisms were amoxicillin, erythromycin, azithromycin, ciprofloxacin, valproic acid and paracetamol in all cities with RCR >1 (Table 4). Ciprofloxacin was dropped off the top priority list when a DF of 40 was applied in Mosul and Basrah and also paracetamol in Baghdad. The highest ranked compounds based on chronic endpoints were amoxicillin, clarithromycin, diclofenac, miconazole nitrate and mefenamic acid at DF = 10 and only amoxicillin showed RCR >10 in Basrah at DF = 40.

In soil, theophylline was ranked highest priority based on the effect on lower trophic level organisms (earthworm). Based on the potential for secondary poisoning in the aquatic environment (i.e. risk to mammalian predators), only phenylephrine had an RCR >1 for all the city scenarios. For secondary poisoning in the terrestrial environments (i.e. earthworm-eating birds and mammals), the highest ranked compound was atropine with an RCR between 0.1 and 1 (Table 5).

**Table 5:** Top 20 compounds from each prioritization approach considered (Baghdad only), according to the predicted concentrations in soil (PECsoil)

RCR	Low level trophic	Higher trophic levels Mammalian predator	
	PECsoil: PNECworm	PECearthworm: PNECmammal	PECearthworm: ADI
>10			
1–10	1 Theophylline 2 Omeprazole 3 Olanzapine		
0.1–<1	4 Fluoxetine 5 Atropine sulphate 6 Guaifenesin 7 Ciprofloxacin 8 Phenylephrine 9 Metoprolol 10 Mefenamic acid 11 Octreotide 12 Procyclidine 13 Valproic acid 14 Dextromethorphan Hydrobromide 15 Pethidine	1 Atropine sulphate	
<0.1	16 Diphenhydramine 17 Sitagliptin 18 Flutamide 19 Trifluoperazine 20 Fluovastatin	2 Procyclidine 3 Olanzapine 4 Diazepam 5 Metoclopramide 6 Octreotide 7 Omeprazole 8 Sitagliptin 9 Guaifenesin 10 Dextromethorphan Hydrobromide 11 Diphenhydramine 12 Metoprolol 13 Ranitidine 14 Chlorphenamine Maleate 15 Ciprofloxacin 16 Theophylline 17 Pseudoephedrine 18 Pethidine 19 Neostigmine 20 Escitalopram oxalate	1 Atropine sulphate 2 Olanzapine 3 Omeprazole 4 Octreotide 5 Procyclidine 6 Metoprolol 7 Escitalopram oxalate 8 Sitagliptin 9 Thyroxine sodium 10 Ranitidine 11 Guaifenesin 12 Dextromethorphan Hydrobromide 13 Trifluoperazine 14 Ketotifen 15 Letrozole 16 Midazolam 17 Metoclopramide 18 Infliximab 19 Pseudoephedrine 20 Bromhexine

*PECsoil, PECearthworm* predicted environmental concentrations in in soil and earthworm, *ADI* acceptable daily intake, *PNECmammal, PNECworm* predicted no-effect concentrations in mammals and in worm

### Comparison of ranking outcomes

Generally, the outcome of the risk-based prioritization showed that the majority of the top ranked pharmaceuticals were antibiotics. Based on all risk comparisons, a final list of 23 compounds (amoxicillin, amitriptyline, ampicillin, atorvastatin, azithromycin, cefalexine, ceftriaxone sodium, ciprofloxacin, clarithromycin, diclofenac, erythromycin, ibuprofen, valproic acid, mebeverine, mefenamic acid, metronidazole, miconazole nitrate, olanzapine, omeprazole paracetamol, phenylephrine, theophylline, trimethoprim) which had an RCR >1 for at least one endpoint or compartment was generated. Interestingly, the results of the current prioritization approach agreed with previously published prioritization studies from other countries. Amoxicillin, the compound with the highest score in this study, was also ranked the top veterinary medicine with high hazard to aquatic organisms in the UK and Korea (Boxall et al. 2003; Kim et al. 2008). Clarithromycin and azithromycin where found alongside amoxicillin on the top priority list in France (Besse and Garric 2008). Paracetamol, amoxicillin and azithromycin were ranked as highly prescribed pharmaceuticals of concern in the USA whereas ciprofloxacin was identified as posing a risk toward aquatic organisms and humans (Dong et al. 2013). Paracetamol, mefenamic acid, amoxicillin, ciprofloxacin, erythromycin and valproic acid were prioritized as highest environmental risk in Switzerland (Perazzolo et al. 2010). A prioritization study performed by Roos et al. (2012) showed amitriptyline, paracetamol, diclofenac and valproic acid to be the highest ranked compounds in one or more comparison studies in Sweden while no antibiotics from this study were found in the ranking lists. Paracetamol ranked the 2nd in terms of usage volume in Sweden while in Iraq it was found to be 1st on the prioritization list. Diclofenac showed a risk score of 0.013 which is equal to the one reported in the UK by Ashton et al. (2004). On the other hand, this compound showed a higher risk score (1–10) in Iraq when chronic ecotoxicological endpoints were used. A recent risk-based prioritization study in the UK has shown most of the antibiotics in our list (amoxicillin, azithromycin, ciprofloxacin, clarithromycin and atorvastatin) to have risk scores greater than 1 in one or more of the risk comparisons proposed (Guo et al. 2016). Amitriptyline was ranked as high priority compound when the potential impact of subtle pharmacological effects was considered by comparing the H_T_PC to estimated levels in fish in the same study. Miconazole was ranked as one of the priority substances used as herd treatment that is moderately used and metabolized (Boxall et al. 2003). It was also found on the top ranking list of pharmaceuticals according to the fish plasma model (Roos et al. 2012). Theophylline showed low risk score in aquatic system, and this agrees with a ranking score of 0.015 in surface water reported by Huschek et al. (2004); while in this study the RCR of theophylline toward terrestrial lower trophic levels was >1 followed by omeprazole and olanzapine. Omeprazole was ranked 19th and 22nd in terms of number of prescribed pharmaceuticals in the prioritization studies in the USA and Sweden (Dong et al. 2013; Roos et al. 2012). No previous prioritization study has ranked phenylephrine as a compound of concern. To our knowledge and after reviewing the literature, antibiotics have not been previously prioritized in surface water in terms of their impact on bacterial community or the susceptibility to pose bacterial resistance.

### Pharmaceuticals of concern on the top of priority lists

Antibiotics are often ranked as the highest priority compounds in risk characterization exercises. Recently, the awareness of the risks of antibiotics in the environment has been raised. For example, the European Environmental Quality Directive has added four antibiotics to the watch list of the Water Framework Directive (Carvalho et al. 2015). All of the added antibiotics (azithromycin, erythromycin, ciprofloxacin and clarithromycin) are ranked as high risk compounds in our priority list. Antibiotics are structurally diverse and do not share a common mode of action (Sanderson et al. 2004), and very low concentrations of antibiotics can be considered extremely harmful to organisms and high concentrations of antibiotics in sediment inhibit the growth of bacteria (Kümmerer 2009a, b).

The occurrence and diverse effects of some of the highly ranked APIs have been reported. Although ciprofloxacin, a fluoroquinolone antibiotic, is highly removed in WWTPs, a concentration of 3.8 μg L^−1^ was detected in wastewater effluent in Australia (Watkinson et al. 2007) and much higher concentrations of 6.5 and 14.0 mg L^−1^ from two lakes and pharmaceutical production effluent in India, respectively (Fick et al. 2009). Ciprofloxacin showed luminescence inhibition to *Vibrio fisheri* at 5 mg L^−1^ of 30-min EC50 (Hernando et al. 2007) and shows high toxicity toward cyanobacteria (*Microcystis aeruginosa*) with an EC50 of 0.005 mg L^−1^ with growth inhibition as the endpoint (Halling-Sorensen 2000). In a recent study, ciprofloxacin exposure resulted in growth inhibition of algae (*Pseudokirchneriella subcapitata*) at a 96-h EC50 of 4.83 mg L^−1^ (Martins et al. 2012). Erythromycin is frequently detected in water around the world with concentrations between 0.13 and 0.89 μg L^−1^ (Meinertz et al. 2011; Hernando et al. 2006). It was found to be toxic to algae using chronic tests with a reported EC50 between 0.01 and 0.1 mg L^−1^ while ecotoxicological results showed that acute toxicity was in the range of 10–30 mg L^−1^ for algae, daphnia and bacteria (Isidori et al. 2005). Clarithromycin, a derivative of erythromycin, was detected in concentrations between 0.01 and 0.54 μg L^−1^ in different countries and has been shown to inhibit the growth of algae and cyanobacteria with EC50 values of 0.0371 and 0.0121 mg L^−1^, respectively (Baumann et al. 2014). The PECsw of amoxicillin, a β-lactam antibiotic, in Iraqi cities was very high and ranged from 0.6 to 24.0 μg L^−1^. This concentration is extremely high in comparison to levels <0.001 μg L^−1^ detected in other countries such as in Italy (Castiglioni et al. 2004). It shows high toxicity to blue-green algae (cyanobacteria) with a reported 96-h EC50 of 0.00222 mg L^−1^ (Fass.se) and is known to cause hepatocyte cytotoxicity as side effect to rainbow trout with a 24-h EC50 >182.7 mg L^−1^ (Laville et al. 2004).

In addition to direct toxicological risks, the occurrence of antibiotics raises concerns in terms of the promotion of antibiotic resistance in bacteria in environment, which could subsequently make antibiotics ineffective in terms of treatment for both humans and animals since aquatic ecosystems are a recognized reservoir for antibiotic-resistant bacteria (Santos et al. 2010; Kostich et al. 2014; Ågerstrand et al. 2015). Interestingly, the occurrence of antibiotic resistance in the environment is not on the main list of priorities that should be addressed by guidelines for the environmental risk assessment of medicinal products for both human and veterinary use in the European Union [European Medicines Agency (EMEA) 2006; 2008]. Studies from different parts of the world have highlighted the fact that resistant strains of bacteria occur in the environment. For example, in Slovakia, the occurrence of resistance to different antibiotics (erythromycin, clarithromycin, azithromycin, ciprofloxacin, trimethoprim) in coliforms and streptococci from WWTPs sludge was studied (Birošová et al. 2014). In Canada, isolated *Escherichia coli* retrieved from different sites and aquatic ecosystem compartments (biofilms, sediment and water) showed high frequency of resistance to ampicillin and ciprofloxacin (Maal-Bared et al. 2013). In Brazil, three strains of *Salmonella* from water samples of a shrimp farm exhibited multiresistance to ampicillin, tetracycline, oxytetracycline and nitrofurantoin (Carvalho et al. 2013). Recently, a study of tetracyclines, sulfonamides and (fluoro)quinolones in sediment and water samples in Guangdong, China, indicated that fish ponds are reservoirs of antimicrobial resistance genes and the presence of potential resistant and pathogen-associated taxonomic groups in fish ponds might imply the potential risk to human health (Xiong et al. 2015).

Two non-steroidal anti-inflammatory drugs (NSAIDs) were identified as high priority APIs i.e. diclofenac and mefenamic acid. In 2013, the European Directive identified diclofenac, alongside two synthetic hormones, as pollutants that should be included in the Water Framework Directive Watch List (Carvalho et al. 2015). van den Brandhof and Montforts (2010) reported the effect of diclofenac on growth retardation in zebrafish after exposure to concentrations >1.5 mg L^−1^. Hoeger et al. (2005) and Schwaiger et al. (2004) documented that diclofenac has the potential to cause histopathological damage to tissues (kidney) in fish at concentrations close to those regularly found in surface waters. Mefenamic acid showed a maximum PECsw (1.2 μg L^−1^) which is higher than the reported levels (0.20-0.34 μg L^−1^) in the UK by Roberts and Thomas (2006). Ecotoxicological effect of mefenamic acid in chronic toxicity tests to *Daphnia magna* and *Moina macrocopa* showed significant changes in reproduction (number of young per adult) after the exposure to 1.0 and 0.25 mg L^−1^ of mefenamic acid, respectively (Jung Collard et al. 2013). The top used compound in Iraq is paracetamol. In our study, the maximum PECsw for paracetamol in Iraqi cities was 23.99 μg L^−1^ in Basrah which is two times higher than the concentration obtained from a study by Jones et al. (2002) who found the maximum PEC in English rivers to be 11.96 μg L^−1^ and more than two orders of magnitude higher than the concentration of 0.11 μg L^−1^ which was detected in 24 % of the rivers in the USA (Kolpin et al. 2002). In terms of ecotoxicological effect, Galus et al. (2013) found that embryonic mortality of zebrafish was raised after exposure to paracetamol at the level of ≥0.5 μg L^−1^. Very limited studies have been performed on ecotoxicity of valproic acid toward environmental organisms. Herrmann (1993) carried out a prescreen test to investigate the possible hazard posed to humans using zebra fish exposure to valproic acid and revealed that exposure caused retardation and interruption of development. The cholesterol-lowering agent atorvastatin was reported to affect lemna (*Lemna gibba*) by decreasing pigment content at EC50 0.17 mg L^−1^ (Brain et al. 2004). It was also found to inhibit growth of *Hyalella azteca* with LC50 values ranging from 1.30 to 3.56 mg L^−1^ and *Chironomus tentans* with LC50 values ranging from 3.94 to 16.42 mg L^−1^ (Dussault et al. 2008). Amitriptyline was identified as a high priority list due to its potential to elicit subtle effect in fish in the current study. It has previously been reported to pose a risk to surface waters and show toxicity to fish and daphnia, EC50 = 0.78 mg L^−1^ (Kasprzyk-Hordern 2010). In lower trophics, amitriptyline was reported to inhibit the growth of a macrophyte *Lemna minor* with a 7-day EC50 of 1.69 mg L^−1^ (Ågerstrand and Rudén 2010).

Ibuprofen is predicted to occur in Iraqi surface water at concentrations of 0.13–0.8 μg L^−1^ and sediment at concentrations of 3.0–20 μg Kg^−1^. The log Kow of 3.73 and low solubility suggest the low mobility of ibuprofen in water and affinity to adsorb to sediment (Bouissou-Schurtz et al. 2014). Ibuprofen was detected at a concentration of 1.3 μg L^−1^ in surface water in Switzerland (Tixier et al. 2003) and 0.15–3.96 μg L^−1^ in the influent and effluent wastewater in Sweden (Bendz et al. 2005). It was found that exposure to chronic low levels of ibuprofen alters the pattern of reproduction of Japanese medaka, *Oryzias latipes*, and may produce sex-specific responses in teleosts (Flippin et al. 2007). Ibuprofen at a concentration slightly higher than 0.2 μg L^−1^ is able to significantly increase both genetic and cellular damage in freshwater bivalve *Dreissena polymorpha* (Parolini et al. 2011).

## Limitation of the method

Knowledge about usage data is essential to establish a priority list of pharmaceuticals of most concern. In Iraq, it was difficult to obtain the consumption amount of all pharmaceuticals from the ministry of health list due to absence of a governmental statistical data and it is sometimes considered confidential. Moreover, it was not possible to quantify the usage data of over-the-counter (OTC) pharmaceuticals. Therefore, an accurate quantification approach of OTC usage should be a future priority. The project did not consider veterinary pharmaceuticals, but this use pattern could also be an important contributor to the environment, particularly for antibiotic compounds.

Another restraint which increases the uncertainty is the limited availability of ecotoxicological endpoints and the high dependence on the prediction of effects and properties. For example, the practice of using ECOSAR to extrapolate ecotoxicity data may not be appropriate since this software was developed to assess toxicity of compounds other than pharmaceuticals. Physico-chemical properties were also limited; for instance, Koc which was used to estimate adsorption during wastewater treatment was calculated by an empirical estimation model (Franco and Trapp 2008) due to absence of experimental values for all the pharmaceuticals on the list. Moreover, bioconcentration factors for worm (BCFworm) was predicted according to the TGD 2003 to allow the secondary poisoning assessment of pharmaceuticals in the terrestrial compartment due to limited availability of experimental data. This estimation is usually higher than the experimentally obtained BCF values (TGD 2003). Therefore, an improvement in the accuracy of BCFworm estimation in soil warrants further consideration.

## Conclusion

An approach has been developed for prioritizing substances that may pose a risk to the aquatic and terrestrial systems in Iraq. Pharmaceutical usage data has been used together with information on the physico-chemical properties, patient metabolism and wastewater treatment removal in this practice to predict API concentrations. The ranking has been performed by comparing these concentrations to a range of experimental and estimated ecotoxicological endpoints including non-standard endpoints such as the potential for subtle pharmacological effects, secondary poisoning and the impact on human via consuming fishery products and drinking water. Dilution factor was found to play an important role to reduce the risk suspected to be posed toward environmental organisms by pharmaceuticals, and results of this study showed that the release of pharmaceuticals to the aquatic environment represents a significant environmental threat, especially when DF is low.

Twenty-three APIs including antibiotics, analgesics, antiepileptics, anti-hypercholesterolemia and anti-asthma have been identified as high priority substances. The study indicates that antibiotics are the pharmaceutical class of most concern with annual consumption of these molecules in Iraq up to 420 t year^−1^. Risks of pharmaceutical compounds in drinking water to human health are low with the exception of tramadol when no WWTP connectivity exists. Large numbers of pharmaceuticals considered in this study could be removed during wastewater treatment, and their risk towards environment will be highly reduced when a proper removal mechanism is used, but in our study case, the removal by this method is neglected due to the absence or inefficient operation of WWTPs in Iraq. Further evaluation is recommended to assess whether these compounds could indeed pose a risk to the environment as individuals or in a mixture since a broad range of different substances is used simultaneously in humans in any given area.

## Electronic supplementary material

Below is the link to the electronic supplementary material.

ESM 1 (DOCX 61 kb).
