# Supplementary material for: Risk-based prioritization of pharmaceuticals in the natural environment in Iraq
Source: Environ Sci Pollut Res Int. 2016 Apr 30;23:15712–26. doi: 10.1007/s11356-016-6679-0 (PMC4956694; doi:10.1007/s11356-016-6679-0)
Supplement: Supplementary file 1 — (DOCX 61 kb). [file 11356_2016_6679_MOESM1_ESM.docx]

**Risk Based Prioritization of Pharmaceuticals in the environment in Iraq**

Omar S. A. Al-Khazrajy^1^ and Alistair B.A. Boxall^1^

1 – Environment Department, University of York, Heslington, Wentworth Way, York, YO10 5NG, UK.

Corresponding author: Alistair B. A. Boxall, Telephone +44 (0)1904 324791; email- alistair.boxall@york.ac.uk

**Supporting information**

$\mathbf{WWinhab=}\frac{\mathbf{Total daily wastewater discharges}}{\mathbf{population of catchment}}$ **(S1)**

Where: Total daily wastewater discharges is the waste water discharge in each of the three cities under study; population of the catchment is the population in each city (Baghdad, Mosul, Basrah).

$\mathbf{Subinhab}\boldsymbol{=}\frac{\mathbf{AP*10^6}}{\mathbf{Iqpop*365}}$ **(S2)**

Where: Subinhab is the amount of substance consumed per inhabitant per day for the Iraqi population [mg inh d^-1^]; AP is the Annual pharmaceutical usage [kg year^-1^]; and Iq_POP_ is Iraq population, which is 34.2 million (COSIT 2012).

$\boldsymbol{PECsed\_ww=}\frac{\mathbf{K susp-water}}{\mathbf{RHOsusp}}\boldsymbol{\times PECwater\times1000}$ **(S3)**

Where: Ksusp-water is the suspended matter-water partitioning coefficient; and RHO is bulk density of suspended matter (Kg m^-3^) (TGD 2003). For the calculation of KSed-water, S4 equation was used.

$\boldsymbol{Ksed-water=Fair\_sed \times Kair-water+Fwater\_sed+Fsolid\_sed \times}\frac{\mathbf{KpSed}}{\mathbf{1000}}\boldsymbol{\times RHOsolid}$ **(S4)**

Default values for Fair_sed, RHOsolid, Fwater_sed and Fsolid_sed were taken from the TGD 2003.

Since the final PECsed was calculated in terms of dry weight, a conversion step was required, using the equations S5 and S6 (Carvalho et al. 2015).

$\mathbf{CONVsed=}\frac{\mathbf{RHOsed}}{\boldsymbol{FsolidSed\times RHOsolid}}$ **(S5)**

$\boldsymbol{PECsed=CONVsed\times PECsed\_ww}$ **(S6)**

Default values for RHOsolid, Fsolid_sed and RHOsed (bulk density of sediment (Kg m^-3^)) were taken from the 2003.

**PECFISH = PECSW * BCFfish * BMF (S7)**

Where PECFISH: Predicted environmental concentration in fish as food [mg kg^-1^];

BMF: Biomagnification factor obtained from the technical guidance document (TGD2003).

$\boldsymbol{BCFFISH= fW\times}\frac{\mathbf{1+}\mathbf{10}^{\mathbf{(pHint-pKa)}}}{\mathbf{1+}\mathbf{10}^{\mathbf{(pHext-pKa)}}}\boldsymbol{+ flip\times Dlip\_water}$ **(S8)**

Where BCFFISH is the Bioconcentration factor for fish; fW is water content fraction of the organism, i.e. 0.7; flip is lipid content fraction of the organism, i.e. 0.1; pHint is internal, i.e. pH, 7.5; and pHext: external pH, 7: Dlip-water is lipid-water partition coefficient, i.e. Log Dlip-water = 0.904 * Log Kow + 0.515, (Fick et al. 2010).

$\boldsymbol{FSSPC = Pblood: water \times PECs}\mathbf{w}$ **(S9)**

Where: FSSPC is the fish steady state plasma concentration [mg L^-1^]; and PECSW is the Predicted environmental concentration for surface water [mg L^-1^]; Pblood: water is the aqueous phase and fish arterial blood partition coefficient.

When

Log KOW <3, **Log Pblood: water = 0.73 * log KOW – 0.88 (S10)**

When

Log KOW >3, **Log Pblood: water = log [(100.73 * log KOW * 0.16) + 0.84] (S11)**

$\mathbf{PECsludge=}\frac{\boldsymbol{Koc \times focsludge \times Subinhab}}{\mathbf{WasteWinhab}}$ **(S12)**

Where PECsludge is Predicted environmental concentration for sludge [mg/kg], ƒoc is organic carbon fraction (0.326).

$\mathbf{PECSOIL=}\frac{\boldsymbol{PECsludge\times Asludge}}{\boldsymbol{Dsoil\times RHOsoil}}$ **(S13)**

Where: PECSOIL is the Predicted environmental concentration for soil [mg kg^-1^]; A sludge is the Sludge application rate to land, i.e. 0.5, [kg m-2 yr^-1^]; DSOIL is the Soil mixing depth, i.e. 0.2, [m]; RHOsoil is the Bulk density of soil, i.e. 1700, [kg m^-3^]; and focSOIL is the Fraction of soil organic carbon, i.e. 0.02.

$\mathbf{C}_{\mathbf{earthworm=}}\frac{\mathbf{BCFearthworm*Cporewater+Csoil*Fgut*CONVsoil}}{\mathbf{1+ Fgut*CONV soil}}$ **(S14)**

Where: Cearthworm (PECearthworm) is the Concentration in earthworm on a wet weight basis [mg kg^-1^]; Cporewater is the Concentration in pore water [mg L^-1^]; C_SOIL_ is the Concentration in soil [mg kg^-1^]; Fgut is the Fraction of gut loading in worm, i.e. 0.1; CONV_SOIL_ is the Conversion factor for soil concentration wet to dry weight soil, i.e. 1.133, calculated from TGD 2003. The BCFearthworm was calculated according to the TGD 2003 approach.

$\mathbf{PECporewater=}\frac{\mathbf{PECsoil}}{\boldsymbol{ƒocsoil\times Koc}}$ **(S15)**

PECporewater: Concentration in pore water [mg L^-1^]; focSOIL: Fraction of soil organic carbon, 0.02.

**BCFearthworm=**$\frac{\mathbf{0.84 * 0.012 Log Kow}}{\mathbf{RHOearthworm}}$ **(S16)**

Where BCFearthworm is the Bioconcentration factor for earthworms [L kg^-1^]; and RHOearthworm is the Density of earthworms (default of 1) [kg L^-1^].

**PNECSW=**$\frac{\mathbf{LC50 or EC50}}{\mathbf{AF}}$ **(S17)**

Where: AF is an assessment factor, (acute QSAR data 1000, acute experimental data 100, chronic QSAR data 100, and chronic experimental data, 10 (TGD 2003).

Sediment water partition coefficient Ksusp-water were derived separately based on Koc and the fraction of organic carbon in sediment (0.05; TGD 2003). Equation (18) was applied to calculate PNECsediment (mgKg^-1^):

$\mathbf{PNECsediment =}\frac{\mathbf{K susp-water}}{\mathbf{RHOsusp}}\boldsymbol{\times PNECwater \times1000}$ **(S18)**

Since the final PNECsediment was calculated in terms of dry weight, a conversion step was also required.

For compounds with no experimentally determined earthworm ecotoxicity data, the terrestrial toxicity (14-d LC50 in mM/kg dry soil) was predicted using the quantitative structure–activity relationship (QSAR) available in ECOSAR using the following equation.

**LogLC50earthworm = 1.405 – 0.308 Log KOW (S19)**

Where:

LC_50 EARTHWORM_: Acute earthworm ecotoxicity, [mM/kg dry soil]

$\mathbf{PNECmammal=}\frac{\mathbf{LD50 (rat or mouse)}}{\mathbf{AF}}$ **(S20)**

$\mathbf{PNEC biota, hh=}\frac{\boldsymbol{0.1 TL\times Bw}}{\mathbf{0.115}}$ **(S21)**

Where: The PNECbiota, hh is the predicted no effect concentration in biota expressed in mg kg^-1^, and uses a default value of human body weight (Bw) of 70 kg, and a daily consumption of fishery products of 0.115 kg. TL, hh, is the acceptable daily intake (ADI). In addition, it is assumed that fishery products make up no more than 10% of the threshold level value (0.1x TL), (EC 2011).

The predicted no effect concentration in drinking water for human (PNECdw, hh) was calculated according to the following equation, retrieved from technical guidance document (EC 2011).

$\mathbf{PNECdw=}\frac{\boldsymbol{0.1 TL\times Bw}}{\mathbf{uptake dw}}$ **(S22)**

A fraction of 0.1 of the human toxicological standard was used, TL hh, the acceptable daily intake (ADI). A human body weight (bw) of 70 kg and a daily uptake of drinking water (uptake dw) of 2 litres were used.

**Table (S1) usage quantities, CAS and therapeutic class of the pharmaceuticals in public health sector and over the counter in Iraq.**

| **compound** | **CAS number** | **Therapeutic class** | **Amount (Kg)**  **over-the-counter** | **Amount (Kg)**  **public health sector** |
| --- | --- | --- | --- | --- |
| Paracetamol | 103-90-2 | Analgesic | 456254.7 | 214708.04 |
| Amoxicillin | 26787-78-0 | Antibiotic | 285607.3 | 134403.395 |
| Metformin Hydrochloride | 1115-70-4 | Antidiabetic | 85810.24 | 40381.284 |
| Cefalexine | 15686-71-2 | Antibiotic | 84515.47 | 39771.98 |
| Metronidazole | 443-48-1 | Antiprotozoal | 83020.64 | 39068.53 |
| Mefenamic acid | 61-68-7 | Anti- inflammatory | 42946.6 | 20210.16 |
| Ibuprofen | 15687-27-1 | NSAID | 30819.52 | 14503.3 |
| Erythromycin | 114-07-8 | Antibiotic | 30339.16 | 14277.25 |
| Trimethoprim | 8064-90-2 | Antibiotic | 27277.55 | 12836.49 |
| Carbamazepine | 298-46-4 | Anti-epileptic | 16617.08 | 7819.8 |
| Ceftriaxone Sodium | 73384-59-5 | Antibiotic | 15204.06 | 7154.85 |
| Guaifenesin | 93-14-1 | Expectorant | 13922.79 | 6551.9 |
| Aspirin | 50-78-2 | Analgesic | 12312.44 | 5794.09 |
| Clarithromycin | 81103-11-9 | Macrolide antibiotic | 12262.31 | 5770.5 |
| Ciprofloxacin | 85721-33-1 | Fluoroquinolone antibiotic | 9740.959 | 4583.98 |
| Ampicillin | 69-53-4 | Beta-lactam antibiotic | 8941.874 | 4207.94 |
| Valproic acid | 99-66-1 | Anti-epileptic | 8862.909 | 4170.78 |
| Theophylline | 58-55-9 | Respiratory diseases | 7933.731 | 3733.52 |
| Ranitidine HCL | 66357-59-3 | Anti-ulcer | 5878.388 | 2766.3 |
| Phenylephrine HCL | 59-42-7 | α1-adrenergic receptor agonist | 4581.182 | 2155.85 |
| Naproxen | 22204-53-1 | NSAID | 3990.007 | 1877.65 |
| Mebeverine HCL | 2753-45-9 | Antispasmodic | 3888.134 | 1829.71 |
| Captopril | 62571-86-2 | Anti-hypertension | 3769.687 | 1773.97 |
| Atenolol | 29122-68-7 | β-blockers | 3584.557 | 1686.85 |
| Diclofenac | 15307-86-5 | NSAID | 3308.009 | 1556.71 |
| Pseudoephedrine | 90-82-4 | Sympathomimetic drug | 3162.851 | 1488.4 |
| Azithromycin | 83905-01-5 | Macrolide antibiotic | 2510.752 | 1181.53 |
| Ceftazidime | 72558-82-8 | Antibiotic | 2471.078 | 1162.86 |
| Diphenhydramine Hydrochloride | 58-73-1 | Antihistamine | 2260.15 | 1063.6 |
| Dextromethorphan Hydrobromide | 125-71-3 | Antitussive | 1878.585 | 884.04 |
| Methyldopa | 555-30-6 | antihypertensive | 1835.575 | 863.8 |
| Losartan Potassium | 114798-26-4 | Anti-hypertension | 1701.807 | 800.85 |
| Miconazole nitrate | 22916-47-8 | Antifungal | 1583.423 | 745.14 |
| Procyclidine HCL | 77-37-2 | Anticholinergic | 1517.42 | 714.08 |
| Amikacin sulphate | 37517-28-5 | Aminoglycoside antibiotic | 1145.481 | 539.05 |
| Cyclosporine | 59865-13-3 | Immunosuppressant | 1035.3 | 487.2 |
| Diltiazem HCL | 42399-41-7 | Calcium channel blocker | 1022.061 | 480.97 |
| Tramadol HCL | 27203-92-5 | Opioid analgesic | 957.6314 | 450.65 |
| Hydrochlorothiazide | 58-93-5 | Diuretic | 925.4376 | 435.5 |
| Mesalazine | 89-57-6 | Anti-inflammatory | 818.8689 | 385.35 |
| Fluovastatin | 93957-54-1 | Anti- hypercholesterolemia | 816.9776 | 384.46 |
| Tetracycline | 60-54-8 | Antibiotic | 797.3214 | 375.21 |
| Hyoscine Butylbromide | 149-64-4 | Antispasmodic | 636.7627 | 299.653 |
| Tranexamic acid | 1197-18-8 | Anti-fibrinolytic | 618.5876 | 291.1 |
| Amitriptyline | 50-48-6 | Anti-depressant | 575.8538 | 270.99 |
| Glibenclamide | 10238-21-8 | Antidiabetic | 563.0188 | 264.95 |
| Lidocaine | 137-58-6 | Antiarrhythmic | 544.7651 | 256.36 |
| Salbutamol | 18559-94-9 | β2-receptor agonist | 460.3388 | 216.63 |
| Acyclovir | 59277-89-3 | Anti-viral | 388.5563 | 182.85 |
| Omeprazole | 73590-58-6 | Anti-ulcer | 365.8188 | 172.15 |
| Atorvastatin | 134523-00-5 | Anti- hypercholesterolemia | 330.7563 | 155.65 |
| Bromhexine HCL | 3572-43-8 | Expectorant | 322.1501 | 151.6 |
| Vancomycin HCL | 1404-90-6 | Antibiotic | 283.69 | 133.5 |
| Chlorphenamine Maleate | 132-22-9 | Antihistamine | 236.385 | 111.24 |
| Levamisole | 14769-73-4 | Anti- cancer | 231.3488 | 108.87 |
| Metoclopramide Hydrochloride | 364-62-5 | Antiemetic | 204.1488 | 96.07 |
| Flutamide | 13311-84-7 | Non-steroidal antiandrogen | 184.365 | 86.76 |
| Amiodarone HCL | 1951-25-3 | antiarrhythmic agent | 168.3213 | 79.21 |
| Sitagliptin | 486460-32-6 | Antidiabetic | 155.55 | 73.2 |
| Nitrofurantoin | 67-20-9 | Antibiotic | 144.0113 | 67.77 |
| Dexamethasone | 50-02-2 | Anti-inflammatory and immunosuppressant | 139.1875 | 65.5 |
| Mycophenolic acid | 24280-93-1 | Immunosuppressant | 119.7225 | 56.34 |
| Diazepam | 439-14-5 | Antianxiety | 115.7488 | 54.47 |
| Furosemide | 54-31-9 | Diuretic | 95.67814 | 45.025 |
| Ifosfamide | 3778-73-2 | Nitrogen mustard alkylating agent (cancer treatment) | - | 42.65 |
| Fluconazole | 86386-73-4 | Anti-fungal | 88.82501 | 41.8 |
| Danazol | 17230-88-5 | Sex hormone | 87.23126 | 41.05 |
| Azathioprine | 446-86-6 | Immunosuppressive | - | 38.81 |
| Phenobarbiton | 50-06-6 | Anti-epileptic | 76.86126 | 36.17 |
| Ketotifen | 34580-14-8 | Antihistamine | 73.58876 | 34.63 |
| Suxamethonium chloride | 306-40-1 | Depolarizing neuromuscular blocker | 70.33751 | 33.1 |
| Bisacodyl | 603-50-9 | laxative | 67.66639 | 31.843 |
| Mesna | 19767-45-4 | Cancer chemotherapy | - | 26.82 |
| Rosuvastatin Calcium | 287714-41-4 | Anti- hypercholesterolemia | 51.94563 | 24.445 |
| Cyclophosphamide | 50-18-0 | Alkylating agent (chemotherapy) | - | 24.19 |
| Chlorpromazine Hydrochloride | 50-53-3 | Anti-psychotic | 45.45376 | 21.39 |
| Teicoplanin | 61036-62-2 | Antibiotic | 43.15876 | 20.31 |
| Pethidine HCL | 57-42-1 | Opioid analgesic | 37.23001 | 17.52 |
| Fluoxetine HCL | 54910-89-3 | Anti-depressant | 28.7725 | 13.54 |
| Olanzapine | 132539-06-1 | Anti-psychotic | 21.52625 | 10.13 |
| Escitalopram oxalate | 128196-01-0 | Anti-depressant | 11.39 | 5.36 |
| Dactinomycin | 50-76-0 | Antibiotic | 7.437501 | 3.5 |
| Ganciclovir | 82410-32-0 | Antiviral | 5.822501 | 2.74 |
| Doxorubicin HCL | 23214-92-8 | Chemotherapy | - | 2.69 |
| Metoprolol | 51384-51-1 | β- blocker | 5.631251 | 2.65 |
| Cerezyme | 143003-46-7 | Antibiotic antineoplastic | 4.250001 | 2 |
| Ondansetron HCL | 99614-02-5 | Gastrointestinal Agent | 3.570001 | 1.68 |
| Trifluoperazine HCL | 117-89-5 | Anti-psychotic | 3.400001 | 1.6 |
| Neostigmine | 59-99-4 | Anesthetic | - | 1.33 |
| Midazolam | 59467-70-8 | Anesthetic | - | 1.31 |
| Bosentan | 147536-97-8 | Antihypertensive | 2.61375 | 1.23 |
| Letrozole | 112809-51-5 | Anti-cancer | - | 0.93 |
| Atropine sulphate | 51-55-8 | Antisialogogue | - | 0.7 |
| Infliximab | 170277-31-3 | Antibody | - | 0.65 |
| Memantine HCL | 19982-08-2 | Anti-Alzheimer's | 1.275 | 0.6 |
| Thyroxine sodium | 51-48-9 | Thyroid Supplement | 0.74375 | 0.35 |
| Epinephrine | 51-43-4 | Hormone | - | 0.27 |
| Octreotide | 83150-76-9 | Growth hormone | - | 0.215 |
| Atosiban | 90779-69-4 | Tocolytic agent | 0.2125 | 0.1 |

**Table (S2) population, wastewater per capita, wastewater treatment percentage and dilution factors for Baghdad, Mosul and Basrah**

| **Catchment**  **(city**) | **Number of population (million)^a^** | **WW_inhab_**  **L/day^b^** | **DF^c^** | **Wastewater treatment %^d^** |
| --- | --- | --- | --- | --- |
| Baghdad | 7.255 | 192 | 10/40 | 50 |
| Mosul | 3.354 | 149 | 10/40 | 0.0 |
| Basrah | 2.602 | 127 | 10/40 | 0.0 |

a: COSIT, 2012; b,d: COSIT, 2014, c: Keller et al. (2014); WWinhab L/day: daily amount of wastewater per inhabitant; DF: dilution factor; Wastewater treatment %: percentage of wastewater treatment plant efficiency

**Table S3 Top 20 compounds from each prioritization approach for exposure via surface water in Basrah at 10 and 40 dilution factors.**

| **RCR** | Low trophic levels | | | | Subtle effects on fish | | Mammalian predator | | Human (uptake from Fishery products) | | Human (uptake from drinking water) |
| --- | --- | --- | --- | --- | --- | --- | --- | --- | --- | --- | --- |
|  | **Acute aquatic** | | **Chronic aquatic** | | **FSSPC: H_T_PC ratio** | | **PECFISH: PNECmammal** | | **PECFISH:PNEC biota, hh** | | **(PECSW: PNECdw, hh)** |
|  | **(PECSW: acute PNECAQUATIC)** | | **(PECSW: chronic PNECAQUATIC)** | |  |  |  |  |  |  |  |
|  | D10 | D40 | D10 | D40 | D10 | D40 | D10 | D40 | D10 | D40 | D10/D40 |
| >10 | Amoxicillin  Azithromycin | Amoxicillin | Amoxicillin  Clarithromycin  Erythromycin | Amoxicillin | Phenylephrine  Atorvastatin  Mebeverine | Phenylephrine  Atorvastatin | Phenylephrine | Phenylephrine | Phenylephrine | Phenylephrine |  |
| 1-10 | Ciprofloxacin  Valproic acid  Erythromycin  Paracetamol  Clarithromycin  Cefalexine | Azithromycin  Ciprofloxacin  Valproic acid  Erythromycin | Diclofenac  Miconazole nitrate  Mefenamic acid | Clarithromycin  Erythromycin | Amitriptyline  Mefenamic acid | Mebeverine |  |  | Atorvastatin |  |  |
| 0.1-1 | Miconazole nitrate  Mefenamic acid  Ibuprofen  Tetracycline  Metronidazole | Paracetamol  Clarithromycin  Miconazole nitrate  Cefalexine  Mefenamic acid  Tetracycline  Ibuprofen  Diphenhydramine | Paracetamol  Azithromycin  Naproxen  Mesalazine  Mebeverine | Diclofenac  Miconazole nitrate  Mefenamic acid  Paracetamol  Azithromycin  Naproxen | Metformin Hydrochloride  Miconazole nitrate  Glibenclamide | Amitriptyline  Mefenamic acid Metformin Hydrochloride | Diazepam  Atorvastatin  Octreotide | Diazepam | Octreotide  Captopril | Atorvastatin  Octreotide | Tramadol |
| <0.1 | Trimethoprim  Atorvastatin  Metformin Hydrochloride  Mebeverine  Glibenclamide  Amitriptyline  Aspirin | Hydrochloride  Atorvastatin  Metronidazole  Procyclidine  Trimethoprim  Amiodarone  Fluoxetine  Diclofenac | Valproic acid  Atenolol  Ranitidine  Ceftazidime  Flutamide  Aspirin  Diazepam  Diltiazem  Nitrofurantoin | Mesalazine  Mebeverine  Valproic acid  Atenolol  Ranitidine  Ceftazidime  Flutamide  Aspirin  Diazepam  Diltiazem  Nitrofurantoin | Amiodarone  Chlorpromazine Hydrochloride  Dextromethorphan Hydrobromide  Ibuprofen  Valproic acid  Diphenhydramine Hydrochloride  Diltiazem  Aspirin  Captopril  Ceftazidime  Diazepam  Metoclopramide Hydrochloride | Miconazole nitrate  Glibenclamide  Amiodarone  Chlorpromazine Hydrochloride  Dextromethorphan Hydrobromide  Ibuprofen  Valproic acid  Diphenhydramine Hydrochloride  Diltiazem  Aspirin  Captopril  Ceftazidime  Diazepam  Metoclopramide Hydrochloride | Miconazole nitrate  Captopril  Ibuprofen  Paracetamol  Azithromycin  Metoclopramide Hydrochloride  Amiodarone  Chlorpromazine Hydrochloride  Mefenamic acid  Amoxicillin  Trimethoprim  Metronidazole  Amitriptyline  Ceftazidime  Dextromethorphan Hydrobromide  Diphenhydramine Hydrochloride | Atorvastatin  Octreotide  Miconazole nitrate  Captopril  Ibuprofen  Paracetamol  Azithromycin  Metoclopramide Hydrochloride  Amiodarone HCl  Chlorpromazine Hydrochloride  Mefenamic acid  Amoxicillin  Trimethoprim  Metronidazole  Amitriptyline  Ceftazidime  Dextromethorphan Hydrobromide  Diphenhydramine | Amiodarone  Ibuprofen  Azithromycin  Miconazole nitrate  Mefenamic acid  Metoclopramide Hydrochloride  Chlorpromazine Hydrochloride  Ranitidine  Ceftazidime  Dextromethorphan Hydrobromide  Amitriptyline  Trimethoprim  Diltiazem  Diazepam  Metronidazole  Amoxicillin | Captopril  Amiodarone  Ibuprofen  Azithromycin  Miconazole nitrate  Mefenamic acid  Metoclopramide Hydrochloride  Chlorpromazine Hydrochloride  Ranitidine  Ceftazidime  Dextromethorphan Hydrobromide  Amitriptyline  Trimethoprim  Diltiazem  Diazepam  Metronidazole  Amoxicillin | Ranitidine  Amoxicillin  Phenylephrine  Paracetamol  Metformin  Trimethoprim  Captopril  Metronidazole  Cefalexine  Atenolol  Valproic acid  Mefenamic acid  Erythromycin  Pseudoephedrine  Theophylline  Ibuprofen  Mebeverine  Clarithromycin |

PECsw: predicted environmental concentration in surface water; FSSPC: fish steady-state plasma concentration; H_T_PC: human plasma therapeutic concentration; PECFISH: predicted environmental concentration in fish; PNEC dw: predicted no-effect concentrations in drinking water; PNECaquatic/PNECmammal: predicted no-effect concentrations in aquatic and mammalian organisms; D: dilution factor.

**Table S4 Top 20 compounds from each prioritization approach for exposure via surface water in Mosul at 10 and 40 dilution factors.**

| **RCR** | Low trophic levels | | | | Subtle effects on fish | | Mammalian predator | | Human (uptake Fishery products) | | Human (uptake from drinking water) |
| --- | --- | --- | --- | --- | --- | --- | --- | --- | --- | --- | --- |
|  | **Acute aquatic** | | **Chronic aquatic** | | **FSSPC: H_T_PC ratio** | | **PECFISH: PNECmammal** | | **PECFISH:PNEC biota, hh** | | **(PECSW: PNECdw, hh)** |
|  | **(PECSW: acute PNECAQUATIC)** | | **(PECSW: chronic PNECAQUATIC)** | |  |  |  |  |  |  |  |
|  | D10 | D40 | D10 | D40 | D10 | D40 | D10 | D40 | D10 | D40 | D10/D40 |
| >10 | Amoxicillin  Azithromycin | Amoxicillin | Amoxicillin  Clarithromycin | Amoxicillin | Phenylephrine  Atorvastatin  Mebeverine | Phenylephrine | Phenylephrine | Phenylephrine HCL | Phenylephrine HCL | Phenylephrine HCL |  |
| 1-10 | Ciprofloxacin  Valproic acid  Erythromycin  Paracetamol  Clarithromycin | Azithromycin  Ciprofloxacin  Valproic acid  Erythromycin | Erythromycin  Diclofenac  Miconazole nitrate  Mefenamic acid | Clarithromycin  Erythromycin | Amitriptyline  Mefenamic acid | Atorvastatin  Mebeverine |  |  | Atorvastatin |  |  |
| 0.1-1 | Cefalexine  Miconazole nitrate  Mefenamic acid  Ibuprofen  Tetracycline  Metronidazole | Paracetamol  Clarithromycin  Cefalexine  Miconazole nitrate  Mefenamic acid | Paracetamol  Azithromycin  Naproxen  Mesalazine  Mebeverine | Diclofenac  Miconazole nitrate  Mefenamic acid  Paracetamol | Metformin Hydrochloride  Miconazole nitrate | Amitriptyline  Mefenamic acid | Diazepam  Atorvastatin  Octreotide |  | Octreotide | Atorvastatin  Octreotide | Tramadol |
| <0.1 | Trimethoprim  Atorvastatin  Metformin Hydrochloride  Mebeverine  Glibenclamide  Amitriptyline  Aspirin | Ibuprofen  Tetracycline  Metronidazole  Trimethoprim  Atorvastatin  Metformin Hydrochloride  Mebeverine  Glibenclamide  Amitriptyline  Aspirin | Valproic acid  Atenolol  Ranitidine HC  Ceftazidime  Flutamide  Diazepam  Diltiazem  Nitrofurantoin  Metformin Hydrochloride | Azithromycin  Naproxen  Mesalazine  Mebeverine  Valproic acid  Atenolol  Ranitidine  Ceftazidime  Flutamide  Diazepam  Diltiazem  Nitrofurantoin  Metformin Hydrochloride | Glibenclamide  Amiodarone  Chlorpromazine Hydrochloride  Dextromethorphan Hydrobromide  Ibuprofen  Valproic acid  Diphenhydramine Hydrochloride  Diltiazem  Aspirin  Captopril  Ceftazidime  Diazepam  Metoclopramide Hydrochloride | Metformin Hydrochloride Miconazole nitrate  Glibenclamide  Amiodarone  Chlorpromazine Hydrochloride  Dextromethorphan Hydrobromide  Ibuprofen  Valproic acid  Diphenhydramine Hydrochloride  Diltiazem  Aspirin  Captopril  Ceftazidime  Diazepam  Metoclopramide Hydrochloride | Miconazole nitrate  Captopril  Ibuprofen  Paracetamol  Azithromycin  Metoclopramide Hydrochloride  Amiodarone  Chlorpromazine Hydrochloride  Mefenamic acid  Amoxicillin  Trimethoprim  Metronidazole  Amitriptyline  Ceftazidime  Dextromethorphan Hydrobromide  Diphenhydramine Hydrochloride | Diazepam  Atorvastatin  Octreotide  Miconazole nitrate  Captopril  Ibuprofen  Paracetamol  Azithromycin  Metoclopramide Hydrochloride  Amiodarone  Chlorpromazine Hydrochloride  Mefenamic acid  Amoxicillin  Trimethoprim  Metronidazole  Amitriptyline  Ceftazidime  Dextromethorphan Hydrobromide  Diphenhydramine Hydrochloride | Captopril  Amiodarone  Ibuprofen  Azithromycin  Miconazole nitrate  Mefenamic acid  Metoclopramide Hydrochloride  Chlorpromazine Hydrochloride  Ranitidine  Ceftazidime  Dextromethorphan Hydrobromide  Amitriptyline  Trimethoprim  Diltiazem  Diazepam  Metronidazole  Amoxicillin | Captopril  Amiodarone  Ibuprofen  Azithromycin  Miconazole nitrate  Mefenamic acid  Metoclopramide Hydrochloride  Chlorpromazine Hydrochloride  Ranitidine HCL  Ceftazidime  Dextromethorphan Hydrobromide  Amitriptyline  Trimethoprim  Diltiazem  Diazepam  Metronidazole  Amoxicillin | Ranitidine  Amoxicillin  Phenylephrine  Paracetamol  Metformin Hydrochloride  Trimethoprim  Captopril  Metronidazole  Cefalexine  Atenolol  Valproic acid  Mefenamic acid  Erythromycin  Pseudoephedrine  Theophylline  Ibuprofen  Mebeverine  Clarithromycin  Amiodarone |

PECsw: predicted environmental concentration in surface water; FSSPC: fish steady-state plasma concentration; H_T_PC: human plasma therapeutic concentration; PECFISH: predicted environmental concentration in fish; PNEC dw: predicted no-effect concentrations in drinking water; PNECaquatic/PNECmammal: predicted no-effect concentrations in aquatic and mammalian organisms; D: dilution factor.

**Table S5Top 20 compounds from each prioritization approach for exposure via surface water in Basrah at 10 and 40 dilution factors.**

| **RCR** | Low trophic levels | | | | Subtle effects on fish | | Mammalian predator | | Human (uptake Fishery products) | | Human (uptake from drinking water) |
| --- | --- | --- | --- | --- | --- | --- | --- | --- | --- | --- | --- |
|  | **Acute aquatic** | | **Chronic aquatic** | | **FSSPC: H_T_PC ratio** | | **PECFISH: PNECmammal** | | **PECFISH:PNEC biota, hh** | | **(PECSW: PNECdw, hh)** |
|  | **(PECSW: acute PNECAQUATIC)** | | **(PECSW: chronic PNECAQUATIC)** | |  |  |  |  |  |  |  |
|  | D10 | D40 | D10 | D40 | D10 | D40 | D10 | D40 | D10 | D40 | D10/D40 |
| >10 | Amoxicillin  Azithromycin | Amoxicillin | Amoxicillin  Clarithromycin  Erythromycin | Amoxicillin | Phenylephrine  Atorvastatin  Mebeverine | Phenylephrine | Phenylephrine | Phenylephrine | Phenylephrine HCL | Phenylephrine HCL |  |
| 1-10 | Ciprofloxacin  Valproic acid  Erythromycin  Paracetamol  Clarithromycin  Cefalexine | Azithromycin  Ciprofloxacin  Valproic acid  Erythromycin  Paracetamol | Diclofenac  Miconazole nitrate  Mefenamic acid | Clarithromycin  Erythromycin | Amitriptyline  Mefenamic acid | Atorvastatin  Mebeverine |  |  | Atorvastatin |  |  |
| 0.1-1 | Miconazole nitrate  Mefenamic acid  Ibuprofen  Tetracycline  Metronidazole  Trimethoprim  Atorvastatin | Clarithromycin  Miconazole nitrate  Cefalexine  Mefenamic acid  Tetracycline  Ibuprofen  Diphenhydramine | Paracetamol  Azithromycin  Naproxen  Mesalazine  Mebeverine | Diclofenac  Miconazole nitrate  Mefenamic acid  Paracetamol  Azithromycin  Naproxen | Metformin  Miconazole nitrate  Glibenclamide | Amitriptyline  Mefenamic acid  Metformin | Diazepam  Atorvastatin  Octreotide  Miconazole nitrate  Captopril |  | Octreotide | Atorvastatin  Octreotide | Tramadol |
| <0.1 | Metformin  Mebeverine  Glibenclamide  Amitriptyline  Aspirin | Atorvastatin  Metronidazole  Procyclidine  Trimethoprim  Amiodarone  Fluoxetine  Diclofenac | Valproic acid  Atenolol  Ranitidine HC  Ceftazidime  Flutamide  Diazepam  Diltiazem  Nitrofurantoin  Metformin | Mesalazine  Mebeverine  Valproic acid  Atenolol  Ranitidine  Ceftazidime  Flutamide  Diazepam  Diltiazem  Nitrofurantoin  Metformin Hydrochloride | Amiodarone  Chlorpromazine Hydrochloride  Dextromethorphan Hydrobromide  Ibuprofen  Valproic acid  Diphenhydramine Hydrochloride  Diltiazem  Aspirin  Captopril  Ceftazidime  Diazepam  Metoclopramide Hydrochloride | Miconazole nitrate  Glibenclamide  Amiodarone  Chlorpromazine Hydrochloride  Dextromethorphan Hydrobromide  Ibuprofen  Valproic acid  Diphenhydramine Hydrochloride  Diltiazem  Aspirin  Captopril  Ceftazidime  Diazepam  Metoclopramide Hydrochloride | Ibuprofen  Paracetamol  Azithromycin  Metoclopramide Hydrochloride  Amiodarone  Chlorpromazine Hydrochloride  Mefenamic acid  Amoxicillin  Trimethoprim  Metronidazole  Amitriptyline  Ceftazidime  Dextromethorphan Hydrobromide  Diphenhydramine Hydrochloride | Diazepam  Atorvastatin  Octreotide  Miconazole nitrate  Captopril  Ibuprofen  Paracetamol  Azithromycin  Metoclopramide Hydrochloride  Amiodarone  Chlorpromazine Hydrochloride  Mefenamic acid  Amoxicillin  Trimethoprim  Metronidazole  Amitriptyline  Ceftazidime  Dextromethorphan Hydrobromide  Diphenhydramine Hydrochloride | Captopril  Amiodarone  Ibuprofen  Azithromycin  Miconazole nitrate  Mefenamic acid  Metoclopramide Hydrochloride  Chlorpromazine Hydrochloride  Ranitidine  Ceftazidime  Dextromethorphan Hydrobromide  Amitriptyline  Trimethoprim  Diltiazem  Diazepam  Metronidazole  Amoxicillin | Captopril  Amiodarone  Ibuprofen  Azithromycin  Miconazole nitrate  Mefenamic acid  Metoclopramide Hydrochloride  Chlorpromazine Hydrochloride  Ranitidine HCL  Ceftazidime  Dextromethorphan Hydrobromide  Amitriptyline  Trimethoprim  Diltiazem  Diazepam  Metronidazole  Amoxicillin | Ranitidine  Amoxicillin  Phenylephrine  Paracetamol  Metformin Hydrochloride  Trimethoprim  Captopril  Metronidazole  Cefalexine  Atenolol  Valproic acid  Mefenamic acid  Erythromycin  Pseudoephedrine  Theophylline  Ibuprofen  Mebeverine  Clarithromycin  Amiodarone |

PECsw: predicted environmental concentration in surface water; FSSPC: fish steady-state plasma concentration; H_T_PC: human plasma therapeutic concentration; PECFISH: predicted environmental concentration in fish; PNEC dw: predicted no-effect concentrations in drinking water; PNECaquatic/PNECmammal: predicted no-effect concentrations in aquatic and mammalian organisms; D: dilution factor.

**Table S6 Top 20 compounds in the three cities from each prioritization approach considered, according to the predicted concentrations in sediment (PECsed) and at 10 and 40 dilution factors.**

| **RCR** | **Baghdad** | | | | **Mosul** | | | | **Basrah** | | | |
| --- | --- | --- | --- | --- | --- | --- | --- | --- | --- | --- | --- | --- |
|  | Acute aquatic  (PECsed: acute PNECsed) | | Chronic aquatic  (PECsed: chronic PNECsed) | | Acute aquatic  (PECsed: acute PNECsed) | | Chronic aquatic  (PECsed: chronic PNECsed) | | Acute aquatic  (PECsed: acute PNECsed) | | Chronic aquatic  (PECsed: chronic PNECsed) | |
|  | D10 | D40 | D10 | D40 | D10 | D40 | D10 | D40 | D10 | D40 | D10 | D40 |
| >10 | Amoxicillin |  |  |  | Amoxicillin  Erythromycin  Azithromycin  Ciprofloxacin | Amoxicillin | Amoxicillin  Clarithromycin |  | Amoxicillin  Erythromycin  Azithromycin  Ciprofloxacin | Amoxicillin  Erythromycin | Amoxicillin  Clarithromycin | Amoxicillin |
| 1-10 | Erythromycin  Azithromycin  Valproic acid  Paracetamol  Ciprofloxacin | Amoxicillin  Erythromycin  Azithromycin  Valproic acid | Amoxicillin  Clarithromycin  Diclofenac  Miconazole nitrate  Mefenamic acid | Amoxicillin  Clarithromycin | Valproic acid  Paracetamol | Azithromycin  Erythromycin  Ciprofloxacin  Valproic acid | Erythromycin  Diclofenac  Miconazole nitrate  Mefenamic acid | Amoxicillin  Clarithromycin  Erythromycin | Valproic acid  Paracetamol | Azithromycin  Ciprofloxacin  Valproic acid | Erythromycin  Diclofenac  Miconazole nitrate  Mefenamic acid | Clarithromycin  Erythromycin |
| 0.1-1 | Cefalexine  Miconazole nitrate  Clarithromycin  Mefenamic acid  Ibuprofen | Paracetamol  Ciprofloxacin  Cefalexine  Miconazole nitrate | Paracetamol  Naproxen  Erythromycin  Azithromycin | Diclofenac  Miconazole nitrate  Mefenamic acid | Cefalexine  Miconazole nitrate  Clarithromycin  Mefenamic acid  Ibuprofen  Tetracycline  Metronidazole | Paracetamol  Cefalexine  Miconazole nitrate  Clarithromycin  Mefenamic acid | Paracetamol  Azithromycin  Naproxen  Mesalazine  Mebeverine | Diclofenac  Miconazole nitrate  Mefenamic acid  Paracetamol | Clarithromycin  Cefalexine  Miconazole nitrate  Mefenamic acid  Ibuprofen | Paracetamol  Clarithromycin  Cefalexine  Miconazole nitrate | Paracetamol  Naproxen  Azithromycin | Diclofenac  Miconazole nitrate  Mefenamic acid |
| <0.1 | Metronidazole  Tetracycline  Trimethoprim  Atorvastatin  Metformin  Mebeverine  Glibenclamide Hydrochlorothiazide | Clarithromycin  Mefenamic acid  Ibuprofen  Metronidazole  Tetracycline  Trimethoprim  Atorvastatin  Metformin  Mebeverine  Glibenclamide  Bromhexine  Hydrochlorothiazide | Mesalazine  Mebeverine  Valproic acid  Atenolol  Ceftazidime  Flutamide  Diazepam  Ranitidine  Nitrofurantoin  Metformin  Dexamethasone | Paracetamol  Naproxen  Erythromycin  Azithromycin  Mesalazine  Mebeverine  Valproic acid  Atenolol  Ceftazidime  Flutamide  Diazepam  Ranitidine  Nitrofurantoin  Metformin  Dexamethasone | Trimethoprim  Atorvastatin  Metformin  Mebeverine  Glibenclamide  Amitriptyline  Metoclopramide | Ibuprofen  Tetracycline  Metronidazole  Trimethoprim  Atorvastatin  Metformin  Mebeverine  Glibenclamide  Amitriptyline  Metoclopramide | Valproic acid  Atenolol  Ranitidine  Ceftazidime  Flutamide  Diazepam  Nitrofurantoin  Metformin  Dexamethasone | Azithromycin  Naproxen  Mesalazine  Mebeverine  Valproic acid  Atenolol  Ranitidine  Ceftazidime  Flutamide  Diazepam  Nitrofurantoin  Metformin  Dexamethasone | Metronidazole  Tetracycline  Trimethoprim  Atorvastatin  Metformin  Mebeverine  Glibenclamide  Bromhexine  Hydrochlorothiazide | Mefenamic acid  Ibuprofen  Metronidazole  Tetracycline  Trimethoprim  Atorvastatin  Metformin  Mebeverine  Glibenclamide  bromhexine  Hydrochlorothiazide | Mesalazine  Mebeverine  Valproic acid  Atenolol  Ceftazidime  Flutamide  Aspirin  Diazepam  Ranitidine  Nitrofurantoin  Metformin | Paracetamol  Naproxen  Azithromycin  Mesalazine  Mebeverine  Valproic acid  Atenolol  Ceftazidime  Flutamide  Aspirin  Diazepam  Ranitidine  Nitrofurantoin  Metformin |

PECsed: predicted environmental concentration in sediment; PNECsed: Predicted no effect concentrations in sediment; D: Dilution factor. The PECsed and PNECsed were calculated with the Equilibrium Partitioning method from the PECsw and PNECsw respectively.

**References**

EC. European Commission, 2003. Technical Guidance Document on Risk Assessment (TGD II) 1–328.

European Commission, 2011. Common implementation strategy for the Water Framework Directive (2000/60/EC). Guidance Document No. 27. Technical guidance for deriving Environmental Quality Standards.

Keller VDJ, Williams RJ, Lofthouse C, Johnson AC (2014) Worldwide estimation of river concentrations of any chemical originating from sewage-treatment plants using dilution factors. Environ Toxicol Chem 33:447–452.

Government of Iraq, central statistics organization COSIT (2012) Environmental Statistics of Iraq for 2011 (in Arabic). <http://cosit.gov.iq/en/env-stats/envstat>. Accessed 1 April 2014

Government of Iraq, central statistics organization COSIT (2014) Environmental Statistics of Iraq for 2013 (in English). <http://cosit.gov.iq/en/env-stats/envstat>. Accessed 15 December 2014
